# Small-Pox in Lahore

**Published:** 1865-05

**Authors:** 


SMALL-POX is ravaging Lahore, 7000 out of a population of 50,000 of the native inhabitants having fallen victims to it within two months. We are not informed what is the state of vaccination in this district.

